# Novel Approaches to Exploiting Invariant NKT Cells in Cancer Immunotherapy

**DOI:** 10.3389/fimmu.2018.00384

**Published:** 2018-03-02

**Authors:** Benjamin J. Wolf, Jiyoung Elizabeth Choi, Mark A. Exley

**Affiliations:** ^1^Agenus Inc., Lexington, MA, United States; ^2^Brigham & Women’s Hospital, Harvard Medical School, Boston, MA, United States; ^3^University of Manchester, Manchester, United Kingdom

**Keywords:** NKT cells, CD1d, iNKT cells, cancer immunotherapy, monoclonal antibody

## Abstract

iNKT cells are a subset of innate-like T cells that utilize an invariant TCR alpha chain complexed with a limited repertoire of TCR beta chains to recognize specific lipid antigens presented by CD1d molecules. Because iNKT cells have an invariant TCR, they can be easily identified and targeted in both humans and mice via standard reagents, making this a population of T cells that has been well characterized. iNKT cells are some of the first cells to respond during an infection. By making different types of cytokines in response to different infection stimuli, iNKT cells help determine what kind of immune response then develops. It has been shown that iNKT cells are some of the first cells to respond during infection with a pathogen and the type of cytokines that iNKT cells make help determine the type of immune response that develops in various situations. Indeed, along with immunity to pathogens, pre-clinical mouse studies have clearly demonstrated that iNKT cells play a critical role in tumor immunosurveillance. They can mediate anti-tumor immunity by direct recognition of tumor cells that express CD1d, and/or via targeting CD1d found on cells within the tumor microenvironment. Multiple groups are now working on manipulating iNKT cells for clinical benefit within the context of cancer and have demonstrated that targeting iNKT cells can have a therapeutic benefit in patients. In this review, we briefly introduce iNKT cells, then discuss preclinical data on roles of iNKT cells and clinical trials that have targeted iNKT cells in cancer patients. We finally discuss how future trials could be modified to further increase the efficacy of iNKT cell therapies, in particular CAR-iNKT and rTCR-iNKT cells.

## Introduction

T cells utilize their unique T cell receptor (TCR) αβ or γδ chain pairs to recognize the universe of antigens. Although many TCRs with extensive somatic V-D-J gene rearrangements recognize peptide antigens within the context of MHC molecules, this is not the only type of antigen that can be recognized. T cells can also utilize near-germline V-J TCR rearrangements to recognize vitamin metabolites, small phosphoantigens, and lipid antigens presented within various highly conserved and non-polymorphic MHC-I like molecules (1–3). Collectively, these non-peptide-recognizing T cells are called “innate-like” T cells and make up a significant proportion of the mammalian T cell compartment (1–4). Importantly, new research is suggesting that these innate-like T cells have important roles in regulating immune reactions not only to pathogens but also to tumors, making them potentially exploitable T cell populations for immunotherapy (1–6). One of the best characterized innate-like T cell subsets that is being leveraged in immuno-oncology are Natural Killer T (NKT) cells, which recognize lipid antigens bound within the antigen presentation molecule CD1d. The best-characterized subset of NKT cells is “invariant” or “iNKT” cells (1–6). Mouse and human iNKT cells are sufficiently conserved that they can respond to each other’s CD1d (7).

## Overview of iNKT Cell Biology

NKT cells are a heterogenous population of innate-like CD1d-restricted T cells, the best known of which are invariant NKT (iNKT) cells (1–9). iNKT cells utilize a near-germline TCRα rearrangement (Vα24-Jα18 in humans and Vα14-Jα18 in mice) combined with a limited TCRβ repertoire (1–4). iNKT cells were originally named because of expression of NK1.1 (CD161C) in some mouse strains (CD161A in humans) but this does not accurately define iNKT cells (5, 6). Instead, iNKT cells are functionally defined by their ability to respond to the lipid antigen α-galactosylceramide (α-GalCer) when bound within CD1d molecules and/or by utilizing monoclonal antibodies against the human invariant TCRα chain (3–8). By utilizing (imperfect) co-expression of NK and T cell markers, absence in CD1d or Jα18 KO mice, CD1d tetramers loaded with α-GalCer, or other methods, iNKTs were discovered to make up a significant proportion of T cells within the mouse liver (~20−50%) and adipose tissue (~10−25%) and are present in significant numbers (~0.5−2%) within the murine thymus, spleen, blood, and bone marrow (3–6). Within humans, iNKT cells are represented at similar frequencies to mice in adipose tissue, but are much less frequent in the liver and other organs where “non-invariant” or “diverse” NKT cells predominate (6, 8, 9). In human peripheral blood, iNKT cells range between undetectable to over 1% of circulating T cells in rare individuals, with a median percentage of approximately 0.05% (6, 8, 9).

**Figure 1:**
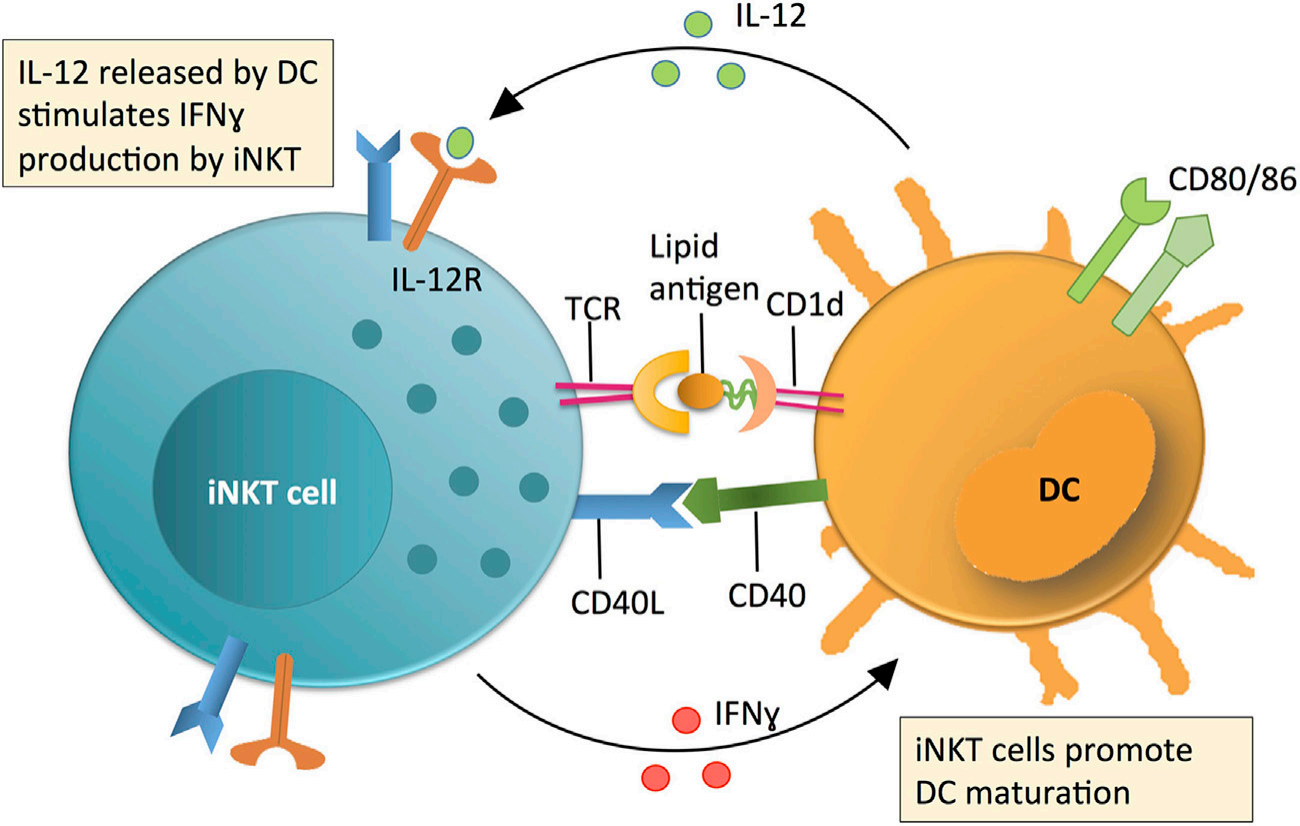
Invariant NKT (iNKT) cell contribution to initiating immune reactions via a positive feedback loop with dendritic cells (DCs). The contribution of iNKT cells to immune surveillance is linked to DC maturation. DCs produce IL-12 and present lipid antigens on CD1d (an MHC class 1 homolog) to stimulate iNKT cell production of IFNγ. The relative contribution of IL-12 or lipid-CD1d to iNKT cell activation is variable and context dependent. Activated iNKT cells produce IFNγ and co-stimulate DCs via CD40L-CD40 interactions to promote DCs to produce IL-12. This IL-12 then further activates iNKT cells in a positive feedback loop. Following activation of iNKT cells, iNKT cell-produced IFNγ and other iNKT-DC interactions (e.g., CD40L-CD40) mature DCs and promote production of IL-12, which further activates iNKT cells in a positive feedback loop.

Unlike peptide-MHC restricted T cells, which emerge from the thymus “naïve,” iNKT cells leave the thymus fully matured and able to perform their effector functions without priming (3–6). Within the periphery, iNKT cells respond to lipid antigen and/or cytokine (e.g., IL-12/18) exposure by rapid secretion of multiple cytokines (3–6). Depending on how the iNKT cells are activated, this can include both regulatory cytokines (e.g., IL-4, IL-10, by analogy with Th2, Treg, etc., especially from NKT2, NKT10) (3–6) and/or pro-inflammatory cytokines (e.g., IL-2, IL-17, TNFα, and/or IFNγ, particularly NKT1 or NKT17) (3–6). Since iNKT cells respond rapidly and without the need for priming, they are some of the first cells within an immune response to be activated and therefore act as a “bridge” between the innate and adaptive immune systems. Indeed, iNKT cell activation via TCR engagement or IL-12 or both causes iNKT cells to upregulate IL-12 receptor [which is already basally expressed at a higher level than in NK cells (6, 8)] and CD40L, while also inducing maturation and production of IL-12 in dendritic cells (DCs). This IL-12 release then in turn greatly increases IFNγ production by iNKT cells, leading to a positive feedback loop for Th1 immunity (Figure 1) (3–6, 8, 9). Additionally, this maturation of DCs leads to trans-activation of NK cells and increased MHC class I and II antigen presentation to T cells as well as direct cognate B cell “help,” allowing for both innate and adaptive immune responses to be established (3–6).

Invariant NKT cells can be further subdivided into additional subsets based on anatomical location or by surface activation markers and transcription factors (1–6). The key master transcription factor of at least iNKT cell development and present in most mature iNKT populations is PLZF (4). Unlike most MHC-restricted T cells that are either CD4 + or CD8α +, iNKT cells in mice are either CD4 + or CD4/CD8αβ double negative (DN) (3–6). In humans, a minor population of iNKT cells (typically 1–5%) can instead express CD8αβ (3, 10). Additionally, CD8αα homodimers are expressed by other activated human T cells, although at lower levels on activated CD4 + T cell subsets lacking CD8β (1, 2, 11). In general, CD4 + iNKT cells are able to express more Th2-related cytokines like IL-4, although they can express as much Th1 cytokines at the same time (3–9). Human CD8αβ + and DN iNKT cells are biased toward a Th1-related phenotype, more cytotoxic and preferentially make IFNγ. In both cases, these are plastic definitions and CD4 + iNKT cells can make IFNγ and DN iNKT cells can make IL-4, at least partially depending on the stimuli given (3–6, 8, 9). Mouse iNKT cells are less clearly biased, although liver iNKT cells have greater anti-tumor activity than other organ iNKT cells (12). Indeed, within different organs, different iNKT phenotypes tend to dominate. Relatively Th1-like iNKT cells tend to be enriched within the spleen and liver, while Th2-like iNKT cells are associated with the lungs and intestine (3–6). There are also Th17-like iNKT cells that express cytokines like IL-17 and are enriched within the lungs, intestine, lymph nodes, and skin (13). Finally, a subset has recently been described in adipose tissue. Adipose iNKT tend to make anti-inflammatory cytokines like IL-10 and unlike other mature iNKT cells, lack PLZF (14, 15).

Because iNKT cells can rapidly produce IFNγ, IL-4, or both, they have been found to play a role in various diseases by establishing a Th1- or Th2-based immune response. In bacterial and viral infections, iNKT cells typically help in early control of the pathogen by establishing a productive Th1 response (1–6, 9, 13, 16). In both mouse and human studies, roles for iNKT cells have been described in diseases associated with excessive Th1 responses like type 1 diabetes (9) and chronic obstructive pulmonary disease (17, 18). Roles have also been described for iNKT cells helping to suppress Th1 responses and drive tolerogenic responses to grafts. As an example, following hematopoietic stem cell transfer, the presence of iNKT cells is predictive for survival with a reduction in graft versus host disease (GvHD) in patients and preclinical models (8, 9, 19–21).

## iNKT Cells in Cancer

Within the context of cancer, the frequency and/or function of iNKT cells (either within the tumor or in circulation) can be selectively and highly correlative with overall survival. In human studies, this has been demonstrated in prostate cancer, medulloblastoma and neuroblastoma, melanoma, colon, lung, breast, and head and neck squamous cell carcinomas (8, 9, 22–37). The largest numbers of patient samples and/or longest follow-ups were analyzed for tumor in neuroblastoma and circulating in head and neck squamous cell carcinomas, respectively (26, 37). Consistent with reduced numbers, proliferative response defects of iNKT cells have been noted in cancer patient iNKT cells (23, 28, 37). Decreased numbers of circulating iNKT cells can be accompanied by decreases in IFNγ production and a concurrent increase in IL-4 production (22, 24, 25, 35). Importantly, all of these defects including the shift toward an iNKT cell Th2 phenotype can be reversed *in vitro*. Activation via 2 strong stimuli, such as α-GalCer and IL-12, increases iNKT cell IFNγ production, promotes tumor rejection, and protects from development of metastasis in multiple mouse models and enhances cancer patient iNKT Th1 responses *in vitro* (8, 22, 24, 25, 35). However, such stimuli do not reverse iNKT defects individually (particularly in advanced disease) (8, 22, 24, 25, 35). Additionally, injection of α-GalCer-pulsed DCs (particularly mature DC) can provide a strong anti-tumor effect (31, 34, 35).

## Role of iNKT Cells in Cancer: Pre-Clinical Mouse Models

While the human data is correlative, the role for iNKT cells in providing tumor surveillance has been well-characterized in mouse models. Examples of iNKT-mediated tumor clearance were demonstrated by the lab of Taniguchi et al. (31, 38) as well as those of Smyth and Godfrey (9, 12). iNKT cells were found to be essential for anti-tumor responses induced by α-GalCer (12, 30, 38). Treatment with carcinogen or transfer of carcinogen-induced tumor cell lines in mice lacking iNKT cells (via TCR Jα18 deletion, Jα18-KO) caused tumors to appear at a much higher frequency than in wild-type (WT) mice (39). Additionally, transfer of iNKT cells into Jα18-KO mice was sufficient to cause protection against tumors to a level like WT mice, unless the iNKT cells came from an IFNγ KO mouse (39). Together, these and other results show that even in the absence of exogenous antigens like α-GalCer, iNKT cells can establish a Th1 response to some tumors and can contribute to tumor clearance (8, 9, 29, 32, 39). Further support for iNKT cell-mediated tumor surveillance was obtained with the spontaneous prostate cancer mouse strain: transgenic adenocarcinoma of the mouse prostate (TRAMP). By back-crossing Jα18-KO to TRAMP mice, Bellone et al. suggested that lack of iNKT cells led to accelerated tumor generation and quicker mortality than was detected in WT TRAMP mice (39), consistent with earlier human *in vitro* data (22). However, more recently, a caveat of studies using the original Jα18 KO mice (38) has come to light, most notably the inability of these mice to express TCR Jα regions past Jα19 (40). This impacts the TCR repertoire of conventional T cells, which could also impact *in vivo* immune responses, so new Jα18 KO mice have been developed that do not share this defect (41, 42).

While some CD1d-expressing tumors can probably cause Th1-biased iNKT cell activation, progressive chronic tumor cell growth can also apparently directly cause Th2-biased iNKT cell activation. By utilizing the same TRAMP prostate cancer model as a source of primary prostate tumors, we demonstrated that CD1d-expressing prostate tumor cells can directly activate iNKT cells, but biased them toward making Th2 cytokines (43). While addition of α-GalCer or IL-12 can usually help bias an iNKT cell toward a Th1 phenotype, neither of these stimuli on their own were enough to reverse the tumor cell driven Th2 bias in iNKT cells. However, pulsing the tumor cells with α-GalCer and adding IL-12 at the same time synergized to allow for IFNγ production to occur (43).

**Figure 2:**
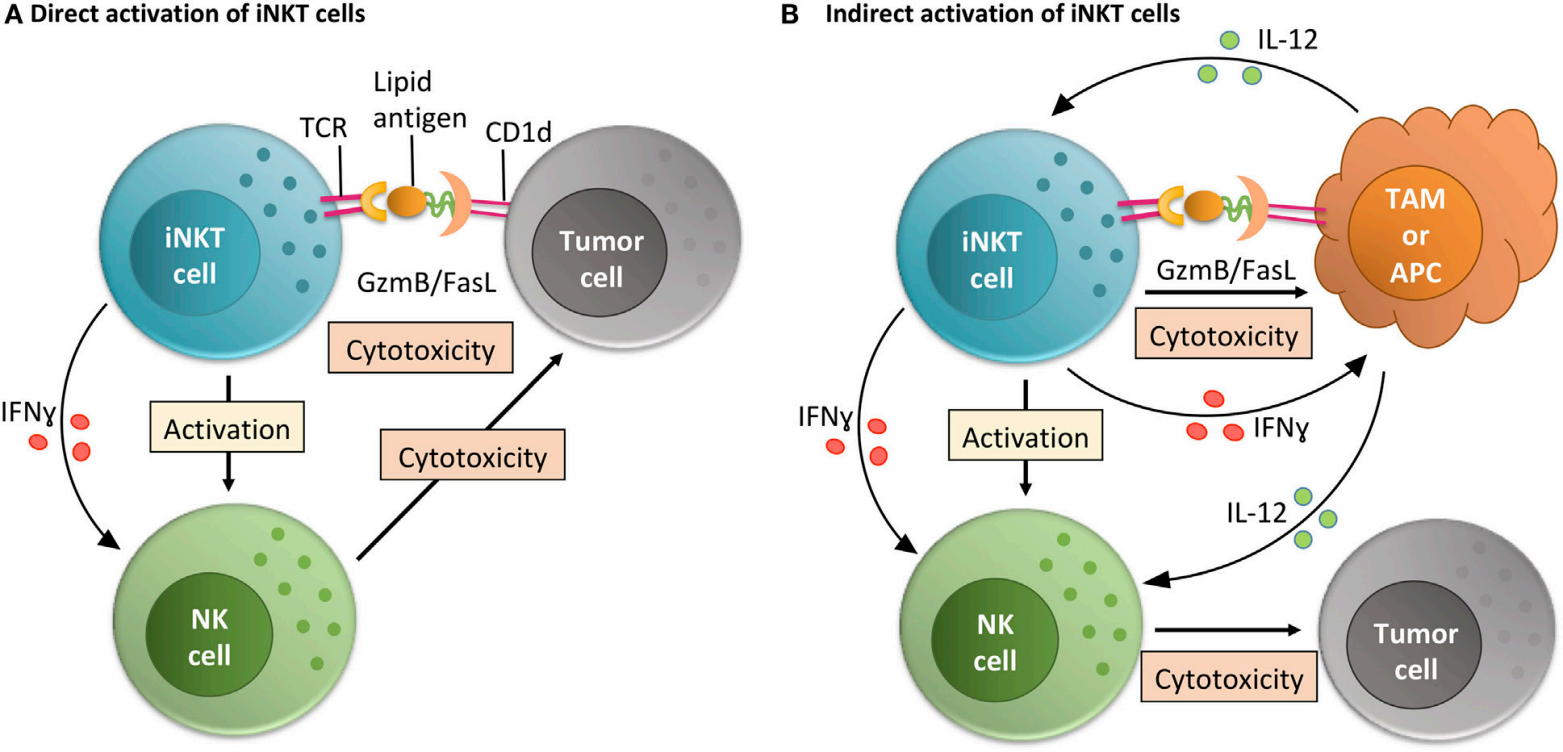
Invariant NKT (iNKT) cell activity within the tumor microenvironment (TME). iNKT cells can function within the TME via direct or indirect interactions with tumor cells. **(A)** In the direct activation pathway, iNKT cells recognize lipid-CD1d complexes on the surface of tumor cells and then directly mediate killing of tumor cells. By making inflammatory cytokines like IFNγ, this also helps TME-resident NK cells perform their anti-tumor cell effector functions (24, 35, 36, 43, 44). **(B)** In the indirect activation pathway, iNKT cells recognize lipid-CD1d complexes on the surface of TME-resident antigen presenting cells (APCs) or tissue-associated macrophages (TAMs) (29). This interaction leads to iNKT cell-mediated killing of immunosuppressive TAMs, leading to a less immunosuppressive environment where tumor-infiltrating NK cells can better perform their functions. Alternately, if the CD1d is on the surface of a TME-resident APC, the iNKT cell can activate that APC and stimulate production of IL-12, helping resident effector cells like NK cells overcome the immunosuppressive state of the tumor (32).

In both the models described above and in humans, activation of iNKT cells and tumor rejection can occur in one of two ways (Figure 2). The first is that iNKT cells directly recognize and kill CD1d-expressing tumor cells. This can occur in a significant portion of lymphomas, early myeloma, myeloid leukemias, medulloblastoma, and prostate cancers (24, 35, 36, 43, 44). The second is by activation of iNKT cells by other CD1d-expressing cells in the tumor microenvironment (TME) (29, 32). In this indirect system, iNKT cell activation by CD1d-expressing TME cells leads to trans-activation of NK cells and/or killing of immunosuppressive cells like tumor-associated Macrophages (TAMs) (29, 32, 45). When we directly tested the ability of iNKT cells to respond to CD1d-expressing prostate tumor cells from TRAMP mice *in vitro*, we found that they did not elicit a Th1 phenotype that would be indicative of killing (43). However, Bellone et al. found that iNKT cells do help delay tumor growth within intact TRAMP mice over periods of months (39). The differences in these two studies may include that within the intact mouse there is also a role for iNKT cells in killing CD1d-expressing TAMs independent of any direct anti-tumor interactions (29, 32). Therefore, relieving some of the immunosuppression within the primary tumor by killing TAMs may be a key role for iNKT cells *in vivo*. However, in progressive clinical cancer, TAMs can apparently overwhelm iNKT cells (29, 32). Reversing these as well as tumor cell-driven iNKT defects is the goal of the groups working on clinical trials targeting iNKT cells worldwide.

## Preclinical and Clinical Trials Targeting iNKT Cells

Pre-clinical murine models have shown similar defects in iNKT cells as have been seen in humans and demonstrated that iNKT stimulation *in vivo* or adoptive transfer can induce strong antitumor immune responses (38, 43, 46–51). This has been shown to be the case for stimulation of iNKT cells via α-GalCer infusion and when α-GalCer has been loaded on DCs (46–51). Additionally, as iNKT cells play a key role in generating a positive feedback loop for IL-12 production by DCs, low and moderate-dose IL-12 therapy in animal models is also dependent on iNKT cells (46, 50, 51). Either stimulation causes iNKT cells to rapidly produce a strong cytokine response, including large amounts of IFN-γ that stimulates NK cells, B cells, and that also enhances the generation of classical cytotoxic T cell responses (39, 49, 51). Strong antitumor immune responses to α-GalCer and/or IL-12 have been observed in most murine models, including colon carcinoma, lymphomas, sarcoma, melanoma, prostate, and lung carcinoma (39, 41, 46–51). Together, these observations indicate that restoration of iNKT cell function in humans with cancer may stimulate potent antitumor immune responses.

### Clinical Trials Targeting iNKT Cells via Stimulation with α-GalCer

The pre-clinical antitumor effects of α-GalCer stimulated a phase 1 clinical study in advanced-stage cancer patients (52). Administration of α-GalCer was not accompanied by dose limiting toxicity. In this phase 1 study, as in other analyses (9, 28, 29, 37), circulating iNKT cell numbers were found to be decreased in cancer patients (52). The relevance of the decreased size of the iNKT cell pool was demonstrated in the same trial as immunological responses to α-GalCer administration (increases in GM-CSF and TNF-α) were only observed in those patients with higher iNKT cell levels comparable to healthy controls (52).

This initial clinical study and preclinical studies outlined above implied that antitumor effects of α-GalCer in cancer patients would be limited by both qualitative and quantitative defects in iNKT cells, necessitating the evaluation of alternative approaches to exploit this natural antitumor system. In mice, administration of α-GalCer-loaded DC resulted in a more powerful antitumor immune response (48, 49). Several phase 1 clinical studies have used α-GalCer-loaded immature or matured monocyte-derived dendritic cells (Mo-DC) or other monocyte-derived antigen-presenting cell (APC) preparations leading to clinically relevant antitumor responses (53–58).

In the first published autologous Mo-DC transfer clinical study, Nieda et al. investigated the transfer of purified α-GalCer-pulsed immature Mo-DC in a variety of different malignancies (53). They found that adoptive transfer of α-GalCer-pulsed Mo-DC led to minor systemic side effects in 9 of 12 patients such as fever, malaise, lethargy, and headache (53). These side effects were temporary and expected when eliciting an immune response by activating iNKT cells. Several patients experienced temporary exacerbation of tumor symptoms that were interpreted as inflammatory responses to the tumor (e.g., enlargement of tumor deposits or associated lymph nodes, bone pain, and respiratory symptoms in subjects with pulmonary metastases) (53). These exacerbated tumor symptoms had a strong temporal and reproducible relationship in terms of timing and nature with treatment cycles, were transient (generally lasting 1 to 3 days), and were absent outside of the study period. In four of the patients, there were decreases in tumor markers, and in one patient, there was extensive tumor necrosis (53). This study was also important in that it was the first to provide clinical interventional data for the role of iNKT cells as the “bridge” between the innate and adaptive immune systems in humans, as has been seen in multiple human *in vitro* and murine *in vivo* studies (39, 43, 46–51). In this clinical trial, activation of human iNKT cells *in vivo* by adoptive transfer of α-GalCer-loaded Mo-DC reproducibly initiated an activation program wherein iNKT cell activation led to subsequent activation of B cells, T cells, NK cells, and increased serum levels of IL-12 and IFN-γ (53).

Ishikawa et al. investigated the effects of adoptive transfer of autologous cell preparations that were enriched for α-GalCer-pulsed DCs in 11 patients with recurrent lung cancer or advanced non-small cell lung cancer (54). No serious adverse events were reported. Importantly, in several patients, an increase in the circulating number of iNKT cells was also detected. Notably, as reported previously, immunological responses were restricted to patients having “normal” pretreatment iNKT cell numbers. No patients exhibited complete or partial responses in this study, but two patients had stable disease (54).

Chang et al. performed a clinical trial where five cancer patients were treated with α-GalCer-pulsed mature Mo-DC (55). The trial was focused on evaluating the number and phenotype of iNKT cells following stimulation via DC transfer. A more than 100-fold expansion of circulating iNKT cell numbers was observed in all five patients, and this expansion was sustained for up to 6 months post-vaccination (55). Additionally, the data suggested a boost in adaptive T cell immunity, as it was accompanied by an increase in antigen-specific memory CD8 + T cells (55). In this study, no more than grade 1 toxicity was observed, and although one patient developed rheumatoid factor and transient positive anti-nuclear antibody at follow up, no clinical evidence of autoimmunity was observed (55).

In addition to these trials above, several subsequent trials have used APC (i.e., adherent monocytic cells treated with GM-CSF and IL-2) loaded with α-GalCer and have shown increasing effectiveness as dose and targeting have been improved, particularly so far in lung cancer and head and neck cancers (56–58). Specifically in a lung cancer trial, patients who had circulating iNKT able to produce IFNγ had a threefold longer lifespan (57).

### Clinical Trials Boosting Endogenous iNKT Cell Numbers via Adoptive Transfer

Another (and complementary) approach to α-GalCer-based treatments involves the adoptive transfer of activated iNKT cells to restore iNKT cell numbers and potentially iNKT cell function in cancer patients. This approach has been tested in preclinical models of melanoma and lung cancer and shown to be more effective compared to the i.v. administration of α-GalCer (50). Trials of iNKT-enriched PBMC have supported direct use of iNKT with evidence for immunological and objective clinical responses (59–62).

The first of these adoptive iNKT cell therapies targeted six patients with non-small cell lung cancer (59). To grow out iNKT cells, bulk PBMCs were stimulated two to three times via addition of α-GalCer to the cultured cells. These iNKT cell-enriched products were then infused back into the patient, and the iNKT cell numbers, persistence, and phenotype were measured. In most patients, there was a transient but not long-term increase in iNKT cell number within the blood, and this coincided with the ability to detect IFNγ production *ex vivo* via α-GalCer stimulation of PBMCs. Only minor adverse effects were seen in this first trial, demonstrating that adoptive cell therapy of iNKT cells is likely to be safe. In this study, no partial or complete responses were seen (59).

The next adoptive iNKT cell-based therapy studies combined autologous iNKT cell-enriched product with *in vivo* boosting. In a Phase I and subsequent Phase II study, the trial group first treated head and neck squamous cell carcinoma (HNSCC) patients with two doses of α-GalCer-loaded DCs followed by an iNKT cell infusion (60). In the Phase I trial, three patients showed partial responses, four had stable disease, and one had progressive disease (60). Of the eight patients, only one had grade 3 adverse events and that patient also had a partial response: a fistula formed within the tumor apparently due to rapid tumor killing (60). In the follow-up Phase II trial for 10 patients with HNSCC, patients were first given nasal submucosal administration of α-GalCer loaded DCs followed by iNKT cell infusion directly into the tumor-feeding arteries, so that iNKT cells were more likely to end up in the tumor site (61). Adverse events were minimal and limited to grade 2 or below, five patients had a partial response, and five patients had stable disease (61). iNKT cell numbers within the tumor and in the peripheral blood were measured, and while iNKT cell numbers in the blood did increase in 9 of 10 patients post-treatment, this did not correlate with outcome. Instead, a high number of tumor-infiltrating iNKT cells correlated with an objective response of patients (61).

With clinical colleagues at Harvard Cancer Center, we performed a Phase I clinical trial of autologous purified [with the iNKTCR mAb 6B11 (62)] and expanded iNKT cells in nine melanoma cancer patients (62). In our study, iNKT cells were isolated from PBMCs with a protocol based on a monoclonal antibody that specifically recognizes the invariant TCR of iNKT cells and then expanded *in vitro* with plate bound anti-CD3 antibody (62–64). Compared to previous studies using α-GalCer stimulated PBMCs as a source of iNKT cells (59–61), this study transferred in generally higher purity and/or larger numbers of iNKT cells (3 doses at up to 250 million iNKT cells per dose). Since iNKT cells are activated via interaction with CD1d on APC, after the first three patients had no significant toxicities, subsequent patients were pre-treated with GM-CSF to enhance DC functions before iNKT infusion cycles 2 and 3. Like in the other studies, we noted a transient increase in circulating iNKT cell numbers following adoptive cell transfer and increased activation of other T cells and myeloid cells in some patients, and toxicities were minor and readily treatable (Grade 1 & 2 only) (62). In terms of responses at the end of the study, three patients had no evidence of disease or stable disease, three eventually progressed and responded to subsequent treatment, and three died of disease (one removed from study after infusions, two at 2 or more years post-treatment). Overall, our trial confirms that iNKT cell adoptive therapy is safe and well-tolerated, but modified treatment regimens are likely required to demonstrate efficacy. These could include further conditioning with stimulations like α-GalCer (on APC or free) and/or IL-12 *in vivo*.

## Future Clinical Trials: CAR-iNKT and rTCR-iNKT

Current clinical trials with either α-GalCer-loaded Mo-DCs or adoptive transfer of iNKT cells have produced partial and complete responses, but few if any cures in late stage patients. In contrast, T cells expressing chimeric antigen receptors (CAR-T) targeting surface proteins like CD19 have shown complete response rates of up to 90% in specific diseases such as B-ALL, leading to the first approvals for these treatments (65). Additional T cell therapies are utilizing recombinant TCR (rTCR-T) expressing T cells to be able to target peptides from tumor-associated intracellular proteins within the context of HLA molecules and are reporting similar complete response rates in myeloma (66) and solid malignancies.

One of the major drawbacks of CAR-T/rTCR-T cell therapy is a very high rate of serious adverse effects, including cytokine release syndrome (CRS) and lethal neurotoxicity (65, 67). Other risks include antigen selection issues (e.g., off-tumor on-target) and GvHD caused by TCR mispairing in rTCR-expressing cells and via allogeneic cell therapy (67). Interestingly, while GvHD is a common concern for both CAR-T and rTCR-T, iNKT cells have been shown in pre-clinical models to suppress, not cause GvHD and are associated with reduced GvHD in the clinic (19–21, 68, 69), making for a potentially safer therapeutic approach. In light of both the promises and drawbacks of CAR-T and rTCR-T cells, there is growing interest in utilizing iNKT cells as an ideal platform for CAR or rTCR therapies (CAR-iNKT or CAR-rTCR).

**Figure 3:**
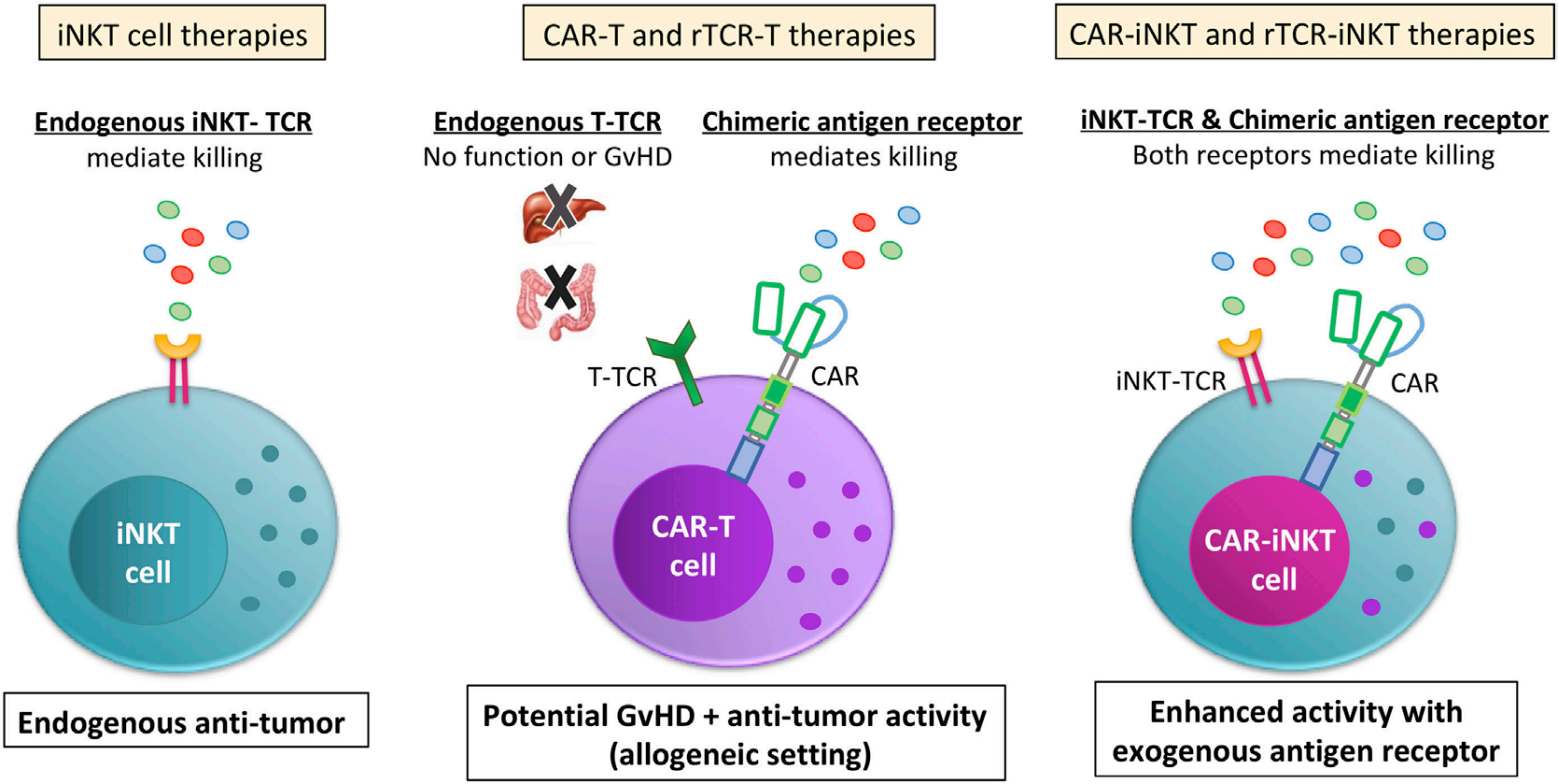
Invariant NKT (iNKT) cells are a viable cell vector for emerging CAR and rTCR therapies. Unlike bulk T cell preparations that are currently used for chimeric antigen receptor (CAR) or recombinant TCR (rTCR) T cell therapies, the endogenous iNKT-TCR has anti-tumor function (left side). In contrast, the bulk T cell endogenous TCRs do not contribute to the anti-tumor function of the cell (middle picture). Instead, the bulk T cell endogenous TCR is a liability, endowing the cells with the potential for graft versus host disease (GvHD) or other off-target effects. While GvHD is a common concern for both CAR-T and rTCR-T allogeneic therapy and at least a possibility due to mispairing for autologous rTCR-iNKT, iNKT cells are shown in pre-clinical models to suppress, not cause GvHD during allogeneic transfer, making for a safer therapeutic approach. Additionally, iNKT cells expressing a CAR or rTCR (right picture) would be endowed with two different anti-tumor receptors. This would allow iNKT cells to target the tumor directly with the CAR or rTCR while its endogenous iNKT-TCR would be able to target the tumor microenvironment and/or the tumor itself, depending on if the tumor expressed CD1d. Either way, CAR-iNKT and rTCR-iNKT therapies would have an endogenous TCR that contributes to tumor clearance instead of be a hindrance.

One of the main benefits of considering iNKT cells as an ideal vector for CAR/rTCR therapies is that iNKT cells have an endogenous TCR that is confirmed to have intrinsic anti-tumor capabilities (Figure 3). While the “random” endogenous TCRs on a bulk polyclonal T cell preparations are unlikely to contribute to anti-tumor effects, it is likely that iNKT cells could utilize both their endogenous TCR and their CAR/rTCR to target the tumor with two different targeting moieties. As mentioned previously, this could be by direct CD1d targeting either on the surface of the tumor cell or a bystander tumor-promoting myeloid cell, or via removing immunosuppression by killing CD1d + TAMs. Since strong TCR signaling cascades (as evidenced by potent iNKT cell antigens like α-GalCer) help cause Th1-based iNKT cell responses, having a second TCR signaling pathway engaged within the iNKT cell may help ensure that the iNKT cells remain Th1-biased *in vivo*. Another advantage for CAR-iNKT or rTCR-iNKT cell therapies is that iNKT cells naturally migrate into non-lymphoid tissues (70), suggesting that they would be ideal cells to target non-lymphoid tumors. Indeed, PLZF expression seems to drive innate T cells tissue homing in general (4, 71). iNKT cells are known to respond to tissue chemokines CCL2 (72) and CCL20 (45). While suggestive of intrinsic benefits of iNKT cells, these points remain to be formally tested in the context of CARs in the clinic.

The Metelitsa group has pioneered CAR-iNKT cells for tumor therapies. In pre-clinical models, they have tested human iNKT cells purified with the iNKTCR mAb 6B11 for their ability to express GD2 CARs (against neuroblastomas) and CD19 CARs (against B cell lymphomas) (73, 74). Importantly, they demonstrated that iNKT cells could stably express either CAR construct, and that the CAR-iNKT cells kill relevant antigen-expressing tumor cell lines *in vitro*. With GD2 CAR-iNKT, this included killing of both GD2 + CD1d− cells and GD2− CD1d + cells, demonstrating that the endogenous iNKT TCR was still functional within GD2 CAR-iNKT cells (73). Importantly, CAR-iNKT cell homing and killing *in vivo* of either the solid tumor xenograft model GD2-expressing neuroblastoma or liquid xenograft B cell lymphoma was greatly increased over non-transduced iNKT cells, leading to substantially increased survival of CAR-iNKT treated mice (73).

As tumor homing and GvHD are concerns for both CAR-T and rTCR-T, they further measured if CAR-iNKT cells had better trafficking to the tumor and what effect placing the CAR into bulk T cells or iNKT cells had on GvHD. CAR-iNKT cells homed to the tumor at an even higher frequency than CAR-T cells, providing evidence that iNKT cells do indeed have better tumor homing than bulk T cells (73). To model GvHD within the context of a xenogeneic cell transfer, CAR-iNKT or CAR-T cells were transferred into humanized mice and monitored for GvHD. As would be expected when transferring in xenogeneic T cells, CAR-T cells caused severe GvHD in the livers and lungs of the mice. In contrast, CAR-iNKT cells did not cause GvHD of these organs during xenogeneic transfer (73), consistent with the GvHD suppressing activities of iNKT described above. Finally, additional work suggested that in GD2 CARs, expression of both the CD28 and 4-1BB costimulatory domains led to longer CAR-iNKT persistence and increased survival of mice compared to single CD28 or 4-1BB costimulatory domains (73). As well as the Metelitsa group, Karadimitris et al. reported in a review otherwise of potential myeloma treatments that CD19-CAR iNKT had promising preclinical anti-tumor activity in their hands also (75). Both groups are planning clinical trials in the near future.

Many groups are looking at transducing subsets of T cells for CAR/rTCR therapies, and some studies have suggested that CD62L+ (central memory) T cells are superior to other T cell subsets (76). In the Metelitsa group CD19 CAR-iNKT study, Tian et al. separated CD62L+ and CD62L− CAR-iNKT cells and measured their persistence and anti-tumor ability (74). CD62L+ CAR-iNKT cells had superior proliferation, *in vivo* persistence, and antitumor activity as compared to the CD62L− CAR-iNKT cells (74), suggesting that even with iNKT cells, it may be worthwhile to target a defined subset for CAR-iNKT or rTCR-iNKT therapies. Interestingly, the majority of iNKT express CD62L until they are repeatedly stimulated *in vitro* (74). While these CAR-iNKT preclinical studies are extremely valuable, critical issues should be generalized within future studies. First, multiple *in vivo* comparisons of tumor killing and survival of CAR-iNKT to bulk T cell CAR-T cells should be performed. Second, as *in vivo* administration of α-GalCer is well tolerated in humans, it should be determined if α-GalCer administration could help either expand CAR-iNKT *in vivo* and/or cause an additive/synergistic increase in anti-tumor activity. Planned clinical studies will begin to address these issues in the near future.

Jiang et al. have provided the first evidence of iNKT cells being able to express a second recombinant TCR (77). In this study, a HLA class I-restricted TCR (TCR-Vα9 TCR-Vβ5) against the *Mycobacterium tuberculosis* (Mtb) 38-kDa protein was cloned and expressed in iNKT cells. Using autologous 38-kDa protein pulsed Mo-DCs as APC, they confirmed that only rTCR-expressing iNKT cells recognized and killed these cells. The relative killing efficiency of α-GalCer-pulsed Mo-DCs was similar to the killing of 38-kDa pulsed Mo-DCs, suggesting that the endogenous iNKT TCR was still fully functional (77). However, it was not determined if the recombinant TCR and the endogenous TCR could both signal at the same time to cause additive or synergistic effects. Nor was it determined if expression of the recombinant TCR came at the expense of some endogenous iNKT-TCR, as could happen due to competition for CD3 complexes. Finally, as it has been reported that the anti-Mtb activity of iNKT cells is due to production of GM-CSF and not production of IFNγ or infected cell lysis (78), it is unclear what additional role(s) rTCR-iNKT cells would play during Mtb infection. Future studies are needed to determine if expressing this rTCR in iNKT cells skews their function *in vivo* during Mtb infection, either by helping trafficking of iNKT cells to the site of infection or otherwise. Clearly, iNKT expressing anti-tumor rTCR could also gain augmented activity, as is currently being addressed by some groups.

## Conclusion

iNKT cells provide a novel alternative to standard T cells in cancer immunotherapy, as described above. Their tissue (and therefore also tumor) tropism, inherent direct and indirect anti-tumor activities and our ability to manipulate them *in vitro* and *in vivo* (e.g., with α-GalCer, analogs thereof, or the iNKTCR mAb 6B11) combined with reversible defects in cancer patients suggest that they can be exploited to treat a range of solid and hematological malignancies.

An important potential caveat in exploiting iNKT cells has been the observation that repeated stimulation of mouse iNKT cells (though less so with human iNKT) with α-GalCer can lead to an anergic-like state (3–6, 8, 79–81). Interestingly, this state can be reversed by PD-1/PD-L1 blockade (79), commonly now used in the clinic to overcome conventional anti-tumor responses. Furthermore, it may reflect a polarization to IL-10 producing “NKT10” that have been found in mice and man (81). Another promising approach in general, which may also overcome NKT cell anergy/polarization, is differential use of co-stimulation alongside direct invariant TCR stimulation (82).

There may well be more total “non-invariant” diverse CD1d-restricted NKT cells in the body than iNKT and their ability to make Th2 cytokines appears to impair tumor immunity (30), whereas such NKT making IFNγ stratifies with cancer patient survival (83), as does iNKT (24, 25, 54). However, non-invariant CD1d-restricted NKT cell manipulation is much more challenging and their understanding lags far behind iNKT cells. Finally, unlike other innate lymphocytes like NK and γδ T cells, iNKT are also relatively rare, so substantially increasing their numbers should be safe and is both very feasible (as described above) and has more potential to change the milieu (the other populations at ~5% of total lymphocytes probably cannot be increased more than ~10-fold without concomitant loss of conventional T cells). The next few years should provide an opportunity for iNKT cells to “put up or shut up”!

## Author Contributions

BW, JC, and ME contributed to the writing and production of this manuscript. JC designed the figures with editorial input from BW and ME.

## Conflict of Interest Statement

BW, JC, and ME were employed by Agenus Inc., a company developing immuno-therapies for cancer, at the time of writing this manuscript.
